# Right pneumonectomy for primary large acinic cell carcinoma (AciCC) with severe mediastinal deviation: a case report and literature review

**DOI:** 10.1186/s12893-021-01351-8

**Published:** 2021-10-18

**Authors:** Xueyu Chen, Yajie Zhang, Fangxiu Luo, Hecheng Li

**Affiliations:** 1 Department of Thoracic Surgery, Ruijin Hospital, Shanghai Jiaotong University School of Medicine, 999 Xiwang Road, Jiading District, Shanghai, 201801 People’s Republic of China; 2 Department of Pathology, Ruijin Hospital, Shanghai Jiaotong University School of Medicine, 999 Xiwang Road, Jiading District, Shanghai, 201801 People’s Republic of China

**Keywords:** Carcinoma, Acinar cell, Lung, Mediastinal deviation, Thoracic surgical procedures

## Abstract

**Background:**

Primary lung acinic cell carcinoma is very rare. Here we report a young female patient who suffered the largest primary lung acinic cell carcinoma with severe mediastinal deviation which has never been reported before. We also reviewed data and features of 20 previously reported cases of primary lung acinic cell carcinoma who underwent lobectomy.

**Case presentation:**

A 27-year-old female patient presented with recurrent coughing and hemoptysis for more than 10 years came to our hospital. A chest computed tomography (CT) showed a giant space-occupying lesion in the hilum of right lung. After a thorough and detailed preoperative examination, the patient then was performed a radical right pneumonectomy with mediastinal lymph node dissection. The size of the tumor was about 8.6 × 4.5 × 4.4 cm. The pathological results demonstrated a primary acinic cell carcinoma of right lung. The immunohistochemistry of the tumor showed AE1/AE3 (+), Ki-67 (2% +), CK7 (+), Vimentin (+), CK19 (+), α1-ACT (+), AB-PAS (+), S-100 (−), TTF-1 (−). The patient was discharged less than 2 weeks after the operation. So far, the patient has been followed-up for 2 years, and no evidence of tumor recurrence or metastasis was observed.

**Conclusions:**

The primary acinic cell carcinoma of lung in this case is the biggest one ever reported and also the first case treated with radical right pneumonectomy. In addition, the patient had a very rare condition of severe mediastinal deviation at the same time. After surgical treatment, the patient recovered uneventfully and had stable disease without recurrence and metastasis after 2 years of follow-up. This case together with the reported case indicate that primary acinic cell carcinoma of lung is of low malignancy, the prognosis and therapy effect of surgical treatment are relatively satisfactory.

## Background

Acinic cell carcinoma (AciCC)is a very rare epithelial malignant tumor of salivary gland, accounting for 10–17% of all malignant salivary gland tumors [1]. It was initially proposed as an independent type of salivary gland tumor by Foote and Frazell in 1953 [2]. In addition to salivary gland, AciCC also has been observed in lung, breast and other organs [3, 4]. Among them, primary lung AciCC is even rare [5] and was firstly reported by Fechner in 1972 [6]. Here we report a case in which the patient suffered a giant AciCC in upper lobe of right lung with severe mediastinal deviation and was treated successfully by surgery.

## Case presentation

A 27-years-old female patient presenting with recurrent coughing and hemoptysis for more than 10 years first came to our hospital in January 2019. According to the medical history information provided, the patient was previously diagnosed as pneumonia and treated symptomatically in local clinical institution. The symptoms of patient were once relieved after the treatment, but recurred frequently. The patient has no family history of primary lung malignancy and genetic disease, 155 cm in height and 42 kg in weight with a lean body shape, symmetrical thorax without deformity. The trachea of the patient deviated to the right slightly, no obvious rhonchi and moist rale as well as wheezing rale were heard. The heart rhythm was regular, the auscultation area of heart sound apparently deviated to the right thorax, and no obvious abnormality was found in abdominal physical examination.

**Fig. 1:**
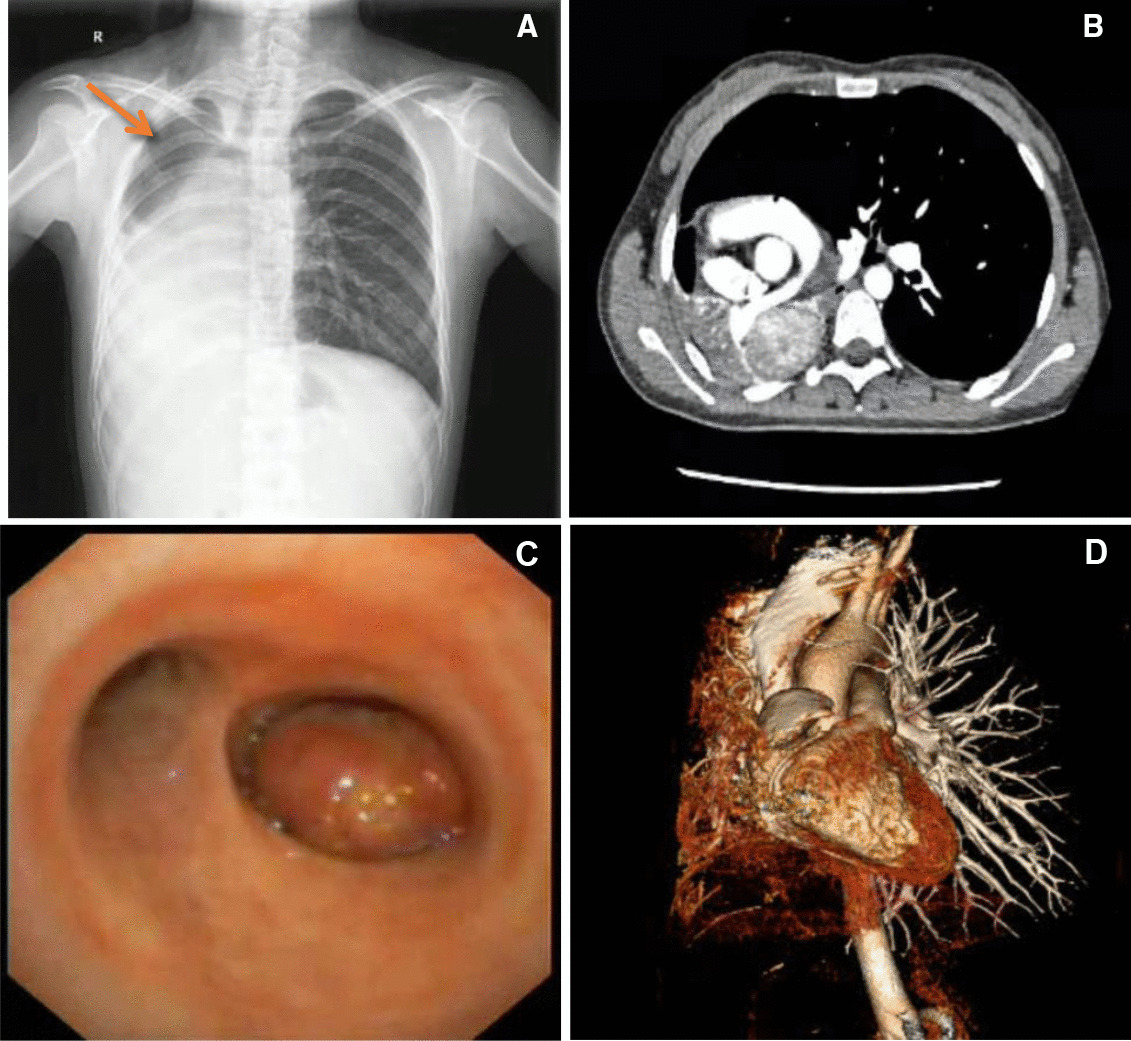
Preoperative imaging of the large acinic cell carcinoma of right lung. **A** Preoperative chest radiograph showed that the mediastinum was completely deviated to the right thorax and the right lung was completely atelectasis. Arrow: part of the left lung tissue that deviate to the right thorax. **B** CT scan showed the right lung was completely atelectated, with tumor tissue invading the right hilum structure. **C** Fiberoptic bronchoscopy revealed a tumor with obstruction of the right main bronchus and protrusion of adjacent organs. **D** 3D-reconstruction showed The vascular anatomical structure of the right hilum is completely disorganized and difficult to recognize

A chest radiograph and computed tomography were performed for the patient showed a giant abnormal space-occupying lesion in the hilum of right lung. The tumor was closely related to the right pulmonary artery and bronchus (Fig. 1a, b). The right lung was atelectasis and some of the left lung as well as heart were obviously deviated to the right thoracic cavity. A Chest MRI showed a mass abnormal signal tumor in the region of right hilum about 10.0 × 4.5 cm in size. The T1WI was isointense, while T2WI and DWI were both hyperintense. A 3D reconstruction of the hilar structures have shown a complete anatomical disorganization of right pulmonary artery and vein (Fig. 1c). A neoplasm with smooth surface was observed in the right main bronchus by bronchoscopy examination. However, a biopsy was not performed concerning that existing hemoptysis might be aggravated (Fig. 1d). Pulmonary function showed a severe obstructive mixed ventilation dysfunction. The forced expiratory volume in 1 s (FEV1) was 1.36 L, accounting for 45% of the predicted value, Maximal voluntary ventilation (MVV) was 27.63L, accounting for 43% of the predicted value. The renal function, electrolyte, coagulation function, arterial blood gas analysis were all normal. Due to long-term persistent hemoptysis, the patient’s blood routine examination showed moderate anemia with hemoglobin 69 g/L. Biochemical examination showed moderate malnutrition, with prealbumin only 85 g/L, albumin 31 g/L. The patient’s tumor markers showed CA125 434.77 µ/ml, CEA 12.43 ng/ml, CA724 233.3 μ/ml, while CA242, CA199, AFP, SCCA, NSE were all normal. No sign of abnormality or metastasis was found in enhanced MRI of brain and bone scan. No abnormal or enlarged lymph nodes were observed in neck and supraclavicular region by ultrasound examination.

We diagnosed the patient with a large tumor in the right lung and firstly considered a special type of malignancy. Because of concerning about a high potential bleeding risk due to the rich supply of tumor and in view of personal willingness of the patient, a preoperative CT-guided percutaneous aspiration biopsy of the tumor was not performed preoperatively. After discussion with a multidisciplinary team which included thoracic surgeons, radiologists, and respiratory physicians, we decided to perform a surgical treatment for the patient.

A thoracotomy was performed under general anesthesia with left lung ventilation. The patient was in left 90°lateral position, a 20 cm long posterolateral incision at the 5th intercostal space was made overlying the right chest wall. Surgical exploration revealed a complete atelectasis of the right lung with obvious consolidation in lung tissue. The lingual segment and anterior segment of left upper lobe herniated into right upper thoracic cavity while the heart of patient also apparently deviated into right lower thoracic cavity. The lymph nodes of each group in mediastinum were checked in the operation and no significantly enlarged lymph nodes were observed. Because of the large size and invasion of the tumor, a right pneumonectomy with mediastinal lymph nodes dissection was initially considered before surgery. The right main bronchus was significantly thicker than normal and the outer diameter of the right main bronchus was 2.4 cm. The bronchial arteries around the bronchus were extremely twisted and dilated. The upper lobe of the right lung was completely consolidated into a mass, the middle and lower lobes of the right lung were atelectatic. Subsequently, we performed a right pneumonectomy for this patient. After confirming the malignacy of the tumor in the right lung and a negative surgical margin by intraoperative frozen section examination, a systematic hilar and mediastinal lymph node dissection was performed.

**Fig. 2:**
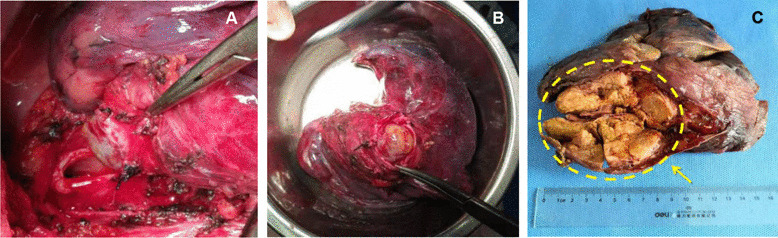
The right lung resected in the operation. **A** The tumor had involved the right main bronchus, the right main bronchus was cut near the tracheal carina to ensure adequate incision margin. **B** The patient’s right lung was radically resected in operation and Intraoperative frozen section of incision margin reported negative. **C** The maximum diameter of the tumor was 8.6 cm, and the tumor tissue extensively invaded into three lobes of patient’s right lung (arrow)

**Fig. 3:**
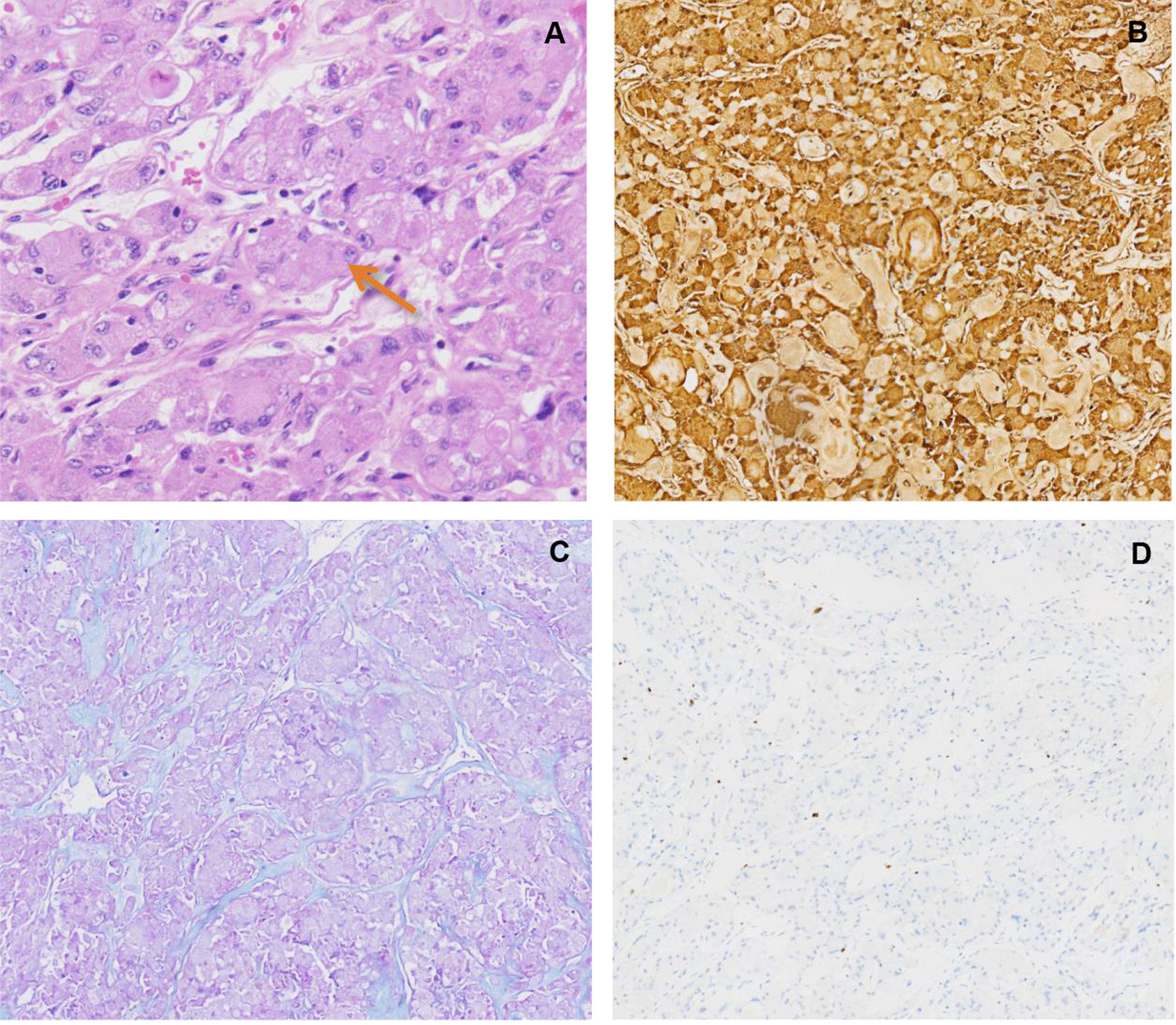
The cellular morphology and immunohistochemistry of the tumour under optical microscope. **A** HE staining shows large, polygonal tumor cells with abundant cytoplasm and aberrant nuclei. The tumor cells are arranged in adenoid and tubular patterns (arrow), × 200. **B** α1-ACT immunohistochemistry staining showed diffuse positive tumor cells, suggesting a certain diagnostic significance, × 200. **C** AB-PAS staining, suggesting that the tumor cells secreted mucus, and the tumor originated from salivary glands, × 200. **D** Ki-67 immunohistochemistry staining showed a low proliferation index, indicating a low grade malignant tumor, × 200

We checked the surgical specimens and found that the tumor originated from the right main bronchus, about 1 cm away from the tracheal carina, grew distally along the lumen of the bronchus and completely blocked the lumen (Fig. 2a, b). The tumor was about 8.6 × 4.5 × 4.4 cm in size (Fig. 2c) and pathological diagnosis showed a malignant tumor orignated from right main bronchus. The cellular morphology of tumor under optical microscope demonstrated that the tumor cells were diffusely arranged and showed large polygonal cells with eosinophilic, granular cytoplasm as well as round, eccentric nuclei. No metastasis of mediastinal lymph node was found. Immunohistochemistry of the tumor showed AE1/AE3 (+), Ki-67 (2% +),α1-ACT (+), AB-PAS (+), CK7 (+), Calponin (±), Vimentin (+), CK19 (+). While CD56, S-100, P63, TTF-1, CDX2, CK5/6, SYN, Dog-1, SOX-10 were all negative (Fig. 3). Combining the microscopic morphology of the tumor cells and the results of immunohistochemical staining, we concluded that this was a case of primary lung AciCC.

The patient recovered well through the operation, only a slight chest tightness appeared in the first few days after the operation, which was quickly relieved after adjustment of drainage. We removed the drainage on the 8th day after surgery and the patient discharged on the 10th day postoperatively. The patient was examined every 3 months during the first year after surgery. Enhanced CT of the chest and abdomen, ultrasound of the superficial lymph nodes, as well as routine blood test, liver function, kidney function, and tumor markers were performed each time. Examinations began semiannually in the second year after surgery, and the contents of the examinations were the same as in the previous year.

At present, the patient has been followed up for 24 months with no evidence of recurrence and metastasis.

## Discussion and conclusions

Primary AciCC of the lung is extremely rare. It is relatively more common in salivary glands tissue. A preoperative examination of the salivary glands was performed in our case and showed no suspicious lesions. There were also no related specific immunohistochemical staining results of salivary gland tissues in postoperative pathology. Therefore, we considered the patient as a primary lung AciCC. A thorough literature search on PubMed shows there were only 20 cases of primary lung AciCC which located in the lower respiratory tract (Table 1). The molecular pathogenesis of primary AciCC still remains unclear. Florian Haller believes that the pathogenesis of the disease may be related to the upregulation of NR4A3 by enhancer hijacking and has important oncogenic functions in AciCC [18]. Primary lung AciCC may occur from juvenile to old age, however there are also two cases reported in young girls younger than 10 years old [12, 17]. The largest tumor size of primary AciCC in lung parenchyma ever reported was 5.2 cm [15], while the size of the tumor in our case is 8.6 cm in maximum diameter, which is larger than that. Meanwhile, it is also rare that the patient in this case had a severe herniation of compensatory left lung expansion and heart which deviated into the right thoracic cavity. Primary lung AciCC is a kind of low-grade malignant tumor that usually presents as an isolated tumor adjacent to or close to the bronchus with little lymph node metastasis. At present, only two scholars, Ukoha and Lee, have mentioned the occurrence of lymph node metastasis in N1 station in their published manuscripts [9, 10].

**Table 1:** Clinical characteristics of primary lung AciCC located in the lower respiratory tract

Case No	References	Publication	Age	Gender	Presentation			Location	Size (cm)	Nodes	Initial	Treatment
		Time (year)			Cough	Hemoptysis	Others				Diagnosis	
1	Fechner et al. [6]	1972	63	M	−	−	−	RL	4.2	N1 (−)	ND	Lobectomy
2	Katz and Bubis [7]	1976	12	F	−	−	Pneumonia	RM + RL	0.8	N1(−), N2 (−)	CCC	Bilobectomy
3	Moran et al. [8]	1992	44	F	−	−	−	RM	3.5	NS	CCC	Lobectomy
4	Moran et al. [8]	1992	48	F	−	−	−	LU	4	NS	OC	Lobectomy
5	Moran et al. [8]	1992	50	F	−	−	−	RM	1.7	NS	ACC	Lobectomy
6	Moran et al. [8]	1992	63	M	+	−	−	RM	1.2	NS	CCC	Lobectomy
7	Moran et al. [8]	1992	75	F	−	−	−	RU	1.5	NS	GCT	Lobectomy
8	Ukoha et al. [9]	1999	63	M	−	−	−	LL	3	N1 (+), N2 (−)	ACC	Lobectomy
9	Lee et al. [10]	2003	30	F	−	−	−	RL	2.1	N1 (+), N2 (−)	ACC	Lobectomy
10	Watanabe et al. [11]	2004	58	M	+	−	−	RM	1.5	NS	ACC	Lobectomy
11	Sabaratnam et al. [12]	2004	4	F	−	+	−	LU	3	N1 (−)	ACC	Lobectomy
12	Vongsivavilas et al. [13]	2005	37	F	−	−	−	RU	3.3	N1 (−)	ND	Lobectomy
13	Cho et al. [14]	2010	68	M	−	−	−	LL	1.8	NS	ND	Lobectomy
14	Papla et al. [15]	2011	63	M	−	−	−	RU + RM	5.2	NS	Liposarcoma	Bilobectomy
15	Zhang et al. [16]	2017	31	M	+	−	Expectoration	RL	4.5	NS	ND	Lobectomy
16	Ling et al. [17]	2019	10	F	+	+	−	LU	1.0, 1.5	N1 (−)	MEC	Lobectomy
17	Ling et al. [17]	2019	25	M	+	−	Chest pain	RU	2.2	N1(−), N2 (−)	ACC	Lobectomy
18	Ling et al. [17]	2019	37	M	−	+	−	RL	1	N1(−), N2 (−)	MEC	Lobectomy
19	Ling et al. [17]	2019	28	M	+	+	−	RM	1	N1 (−)	ACC	Lobectomy
20	Ling et al. [17]	2019	53	F	−	+	−	RM	1.5	N1 (−)	PAC	Lobectomy
21	Our case		27	F	+	+	Pneumonia	RU	8.6	N1(−), N2 (−)	ND	Pneumonectomy

*M* male, *F* female, *CCC* clear cell carcinoma, *GCT* granular cell tumor, *PAC* pulmonary adenocarcinoma, *MEC* mucoepidermoid carcinoma, *OC* oncocytic carcinoma, *LU* left upper lobe, *LL* left lower lobe, *RL* right lower lobe, *RM* right middle lobe, *RU* right upper lobe, *ND* not diagnosed, *NS* not stated

Generally, the surface of tumor is covered with normal tracheal mucosa, it is difficult to get cytological diagnosis by routine bronchoscopy brush examination and biopsy [19]. Through a review of the literature, we found that only less than half of the cases received a definite pathologic diagnosis before surgery. Meanwhile, Kazuo Watanabe [20], a Japanese scholar, believes that transbronchoscopic fine-needle aspiration (FNA) may help to obtain effective samples of tumor tissue to make a definite pathological diagnosis preoperatively. In some cases patients have a history of inhaling foreign bodies and the incidence of primary lung AciCC of right lung is higher than left. Thus, some scholars speculate that the occurrence of primary lung AciCC may also be related to long-term chronic stimulation of inhaling foreign bodies [17].

Surgical resection of the tumor is the main therapy to treat primary lung AciCC. According to the review of available literatures, lobectomy is the main surgical treatment strategy which was adopted and performed in all cases. Among them, two cases received bilobectomy. The tumor in the first case was relatively small. However, due to the close location of tumor from the main bronchus which was less than 1.0 cm, a bilobectomy of right middle and lower lobe was performed inevitably [7]. The other case was performed in 2009 and the tumor was 5.2 cm in maximum diameter. During surgery the tumor was removed together with part of the upper and middle right lobes [15]. In our case, due to the extensive invasion of the tumor into the patient's right lung and its proximity to the right main bronchus, a right pneumonectomy has been a preferable and inevitable surgical treatment strategy to ensure a negative margin of bronchus. The mediastinal and hilar lymph node metastasis rate in patients with primary lung AciCC is relatively low, only Ukoha and Lee reported lymph node metastasis in N1 station [9, 10]. However, some studies showed that the expression of Ki67 antigen is related to the recurrence of the disease. The tumor have an increased likelihood of recurrence when Ki67 > 10%, and mediastinal lymph nodes metastasis can also be found in patient with tumor recurrence [21]. Therefore, we performed systematic lymph node dissection in the operation for this patient and the pathological results showed that all N1 station and N2 station lymph nodes were negative.

Although the size of tumor in this case in very large, the existing literatures does not provide a theoretical evidence for the apparent benefit of postoperative adjuvant radiotherapy and chemotherapy in patients with primary lung AciCC. So far, the patient in this case has been followed up for 2 years after operation without any subsequent adjuvant therapy. No obvious sign of tumor recurrence and metastasis has been observed and all the clinical examination and physical condition are normal. To the best of our knowledge, this is the first case of a primary lung AciCC who underwent right radical pneumonectomy and it is also the biggest tumor ever been reported. This illustrative case demonstrates that the prognosis and surgical therapy effect of primary lung AciCC is relatively satisfactory. However, a further long-term follow-up is still under observation.

## Data Availability

All data generated or analyzed during this manuscript are included in this published article.

## Abbreviations

AciCC: Acinic cell carcinoma
FNA: Fine-needle aspiration
LNs: Lymph nodes
FEV1: Forced expiratory volume in 1 s
MVV: Maximal voluntary ventilation
CEA: Carcinoembryonic antigen
AFP: Alpha-fetoprotein
NSE: Neuron-specific enolase
SCCA: Squamous carcinoma cells antigen
